# Cervical Cancer Screening among Women Receiving Antiretroviral Therapy in a Resource-Limited Environment

**DOI:** 10.31557/APJCP.2020.21.7.2035

**Published:** 2020-07

**Authors:** Moses New-Aaron, Jane L Meza, Martha H Goedert, Stephen M Kibusi, Mkhoi L Mkhoi, Caroline Damian Mayengo, James Charles, Siraji Shabani, Kelsie M Musil, Anlan Cheney, Samwel Sumba

**Affiliations:** 1 *Department of Environmental Health, Occupational Health and Toxicology, University of Nebraska Medical Center, Omaha, Nebraska, USA. *; 2 *Department of Biostatistics, University of Nebraska Medical Center, Omaha, Nebraska, USA. *; 3 *College of Public Health, University of Nebraska Medical Center, Omaha, Nebraska, USA. *; 4 *College of Health Sciences, Public Health, The University of Dodoma, Dodoma, Tanzania. *; 5 *University of Dodoma, College of Health Sciences, Department of Microbiology and Immunology, Dodoma, Tanzania. *; 6 *Ministry of Health, Community Development, Gender, Elderly & Children, Dodoma, Tanzania. *; 7 *Dodoma Regional Referral Hospital, Dodoma, Tanzania. *; 8 *Department of Epidemiology, College of Public Health, University of Nebraska Medical Center, Omaha, Nebraska, USA. *; 9 *Tanzania Commission for AIDS, Tanzania.*

**Keywords:** Cervical cancer screening, knowledge, awareness, willingness, uptake, women living with HIV, prevention

## Abstract

**Background:**

Cervical cancer is among the most prevalent cancer among women worldwide and women living with HIV are at increased risk, especially in a resource-limited environment.

**Objective:**

This study aimed to determine levels of awareness, knowledge, uptake, and willingness to screen for cervical cancer among women receiving care in an HIV clinic at Dodoma Regional Referral Hospital (DRRH), Tanzania.

**Methods:**

Data were collected for a period of three weeks from July 21 to August 11, 2017 using a mobile phone data collection App. A total of 421 Women aged 18-50 years old were included in the study.

**Results:**

Majority of the women interviewed (n=306, 73%) were aware of cervical cancer. Among those who were aware, 84% (n=257) did not recall ever being screened for cervical cancer, and majority had a poor knowledge of cervical cancer. Educational level completed (p=0.01), income per month (p=0.02), age group (p<0.0001), and area of residence (p<0.0001) were all significantly associated to awareness of cervical cancer. Most of the women who have never screened (n=231, 91%) expressed willingness to be screened. Prior uptake of cervical cancer screening was associated with number of live births (p=0.001) and area of residence (p=0.04). And Willingness to screen was significantly associated with age groups (p=0.03) and the number of live births (p=0.03). Moreover, we found that younger age and urban residence was positively associated with awareness and uptake of cervical cancer screening. Willingness was found to decrease as age increased.

**Conclusion:**

The study found that despite older women’s higher risk of cervical cancer, those who indicated willingness to screen were younger. Additional education, health promotion, and integration of cervical cancer screening services is needed to improve cervical cancer awareness and screening uptake at the HIV clinic.

## Introduction

Approximately 60% of all adults living with HIV in Sub Saharan Africa (SSA) are women (Redfield et al., 2019) and the incidence of cervical cancer among women with HIV is higher than women without HIV (Gaffing and Gupta, 2016). In 2012, 527,000 new cases of cervical cancer were reported globally with an estimate of 93,000 new cases and 57, 000 deaths in SSA (Del et al., 2015). By the next decade, experts revealed that approximately 500,000 mortality will be recorded annually in the world due to cervical cancer with most of the cases in SSA (Mboumba et al., 2017). Considering the elevated rates of cervical cancer in SSA (Vaccarella et al., 2013; Mboumba et al., 2017; Jedy-Agba et al., 2020), with about half of the cases in East Africa (GLOBOCAN, 2017) and Tanzania a leading country in terms of incidence and mortality (Kafuruki et al., 2013; Afri-Dev.Info, 2017), it was imperative to conduct this study. 

Generous donations by different supporting partners around the globe have directed resources to endemic areas to combat the HIV threats to lives and livelihoods. These donations strengthened HIV care systems in SSA and, as a result, longevity and quality of life has increased among people living with HIV (PLWH) (Biesma et al., 2009). In 2008, the United States President’s Emergency Plan for AIDS Relief (PEPFAR) program committed $48 billion, which is renewable every 5 years, to address HIV, malaria, and tuberculosis (TB) in SSA (Moss 2008). The Global Fund for AIDS, TB and Malaria (GFATM) (Bennett et al.,2003), The World Bank-supported Multi-country AIDS Program, and The William J. Clinton Foundation (WJCF) (Somi et al., 2009) are other funding partners involved in HIV care and management in SSA. The funds provided specifically for HIV care are channelled towards HIV testing, procurement of antiretroviral drugs (ARVs) and monitoring the progression of patient’s care (Njuguna et al., 2018), but none of these funds provide for cervical cancer screening or education. 

One of the beneficiaries of these donations is The Republic of Tanzania, which has implemented them through a care platform called the HIV Care and Treatment Center (CTC). These centers were established in government-owned hospitals across Tanzania to provide adequate care for PLWH in each region. This study was conducted at the HIV CTC in Dodoma, at the Dodoma Regional Referral Hospital (DRRH), Tanzania. The HIV CTC in DRRH has an extensive capacity and mechanism for dispensing ARVs to patients, however, there are currently no available infrastructures or materials for screening services and education for women living with HIV (WLWHIV) . 

Previous studies have shown that WLWHIV have a higher risk of developing cervical cancer (Grellier and Quéro 2014; Bansil et. al; 2015; Champman et al., 2016; Rohner et al., 2020). In response to this increased risk, cervical cancer prevention among women with HIV is a priority designated at all levels of health governance, and HIV management protocols are beginning to integrate cervical cancer screening services as an important component of care and treatment to WLWHIV (WHO, 2000, Ezechi et al., 2014, Kapambwe et al., 2015). While cervical cancer screening is critical to prevent late presentation of cervical cancer and the increased risk of mortality at later stages (Mukama et al., 2017), integrated screening services are not fully implemented in most HIV clinics across Africa (Kumakech et al., 2015). 

To demonstrate the need for cervical cancer screening among HIV patients, we decided to explore the screening rates among WLWHIV at DRRH. There was only one screening center for cervical cancer to accommodate all women visiting DRRH, which was in the outpatient department of the hospital. This screening center utilizes the visual inspection with acetic acid (VIA) for the basic cytological test of cervical cancer. VIA is the recommended method for screening cervical cancer in Tanzania (TMHSW, 2010) and in other resource-limited environments. This method is limited, however, because confirmatory tests are required to make a complete diagnosis. Participation of VIA screening has also been very low in DRRH for PLWH. The screening rates for women who are at least 18 years old receiving care at the HIV CTC found in the outpatient screening register of DRRH register was about 1% between January 2014 and July 2017.

One of the challenges in the screening protocol of the patients at HIV CTC in DRRH is that suspected cases of cervical cancer using VIA are referred to Ocean Road Cancer Institute in Dar es Salaam which is over 440 km away from Dodoma region. This can have a prohibitive impact on the patient’s decision to participate in the screening at HIV CTC in DRRH for fear of separation from their family and livelihood. Furthermore, educational materials on cervical cancer and the importance of screening were unavailable at the HIV clinic at the time of this study. It was therefore hypothesized that the low screening rates of patients receiving care at HIV CTC were due to lack of awareness and willingness to screen for cervical cancer. In this study, we aimed to determine:

1. the level of awareness, knowledge, and uptake of cervical cancer screening among women receiving ARV treatment from January 2005 to July 2017 and to identify the characteristics of patients who were screened at any time within this period at HIV CTC, DRRH.

2. the prevalence of WLWHIV willing to be screened for cervical cancer at HIV CTC, DRRH.

3. the factors associated with the willingness to be screened, and the uptake of cervical cancer screening among women receiving ARV treatment in HIV CTC, DRRH.

## Materials and Methods

*Methods*

A cross-sectional study was performed using a validated questionnaire (Ezechi et al., 2013, Getahun et al., 2013, and Sichanh et al., 2014). The questionnaire was reviewed by relevant experts (biostatistician, health promotion expert and an epidemiologist), the Institutional Review Board of the University of Nebraska Medical Center (UNMC) and the University of Dodoma (UDOM), Tanzania. Approval to conduct the study was received from IRB of UNMC and UDOM and all participants gave their informed consent before the commencement of the study. During the wait time of participants visiting the HIV CTC, those who gave consent were briefly interviewed at random to determine study participation eligibility. Patients who met the eligibility criteria were assigned the questionnaire. A total of 458 women were accepted initially for the study. Thirteen women who were travelling through Dodoma and needed to visit HIV CTC for drug refills were excluded. Twenty-four women were older than 50 years and were also excluded. As a result, a total of 421 women who were eligible participants were included in the study. 

*Inclusion criteria*

Women between the ages of 18 and 50 years and receiving care at the HIV CTC between January 2005 and July 2017 were eligible for this study.

*Exclusion criteria*

Women who were traveling through Dodoma and needed to visit HIV CTC DRRH for refills were described as patient in-transit and were excluded from the study. 

*Data collection*

Data were collected for a period of three weeks from July 21 to August 11, 2017. The questionnaires were designed on an Open Data Kit (ODK) with a platform called Kobo Toolbox (Heunis et al., 2014) installed on Android mobile phones. Participants with good knowledge of Android phone answered the questions themselves with minimal supervision. Participants without good knowledge of Android phone or were illiterate were interviewed by the research assistants. The majority (n=4, 80%) of research assistants were females. The average time of an interview was 20 minutes per patient while using the mobile phones and 30 minutes for participants who were assisted by a research assistant. All questions were developed in the English language and translated into Swahili by the chief physician of the HIV clinic. The questionnaire was reviewed by the research assistants who understood English and were native speakers of Swahili to validate the translation. 

*Description of outcome variables*

Awareness of cervical cancer was determined by asking if the women had ever heard of cervical cancer. Those who had heard of cervical cancer responded, “Yes,” and those who had not, responded, “No.”

Prior uptake of cervical cancer screening was defined by women who heard of cervical cancer and had been screened either in DRRH or outside DRRH at least once in their lifetime. Participants with prior uptake of cervical cancer screening responded, “Yes,” and those who had not been screened but were aware of cervical cancer responded, “No.”

Willingness to be screened was defined by positive response to the question, “Do you want to screen?” among women who had never been screened for cervical cancer at any point in their lifetime.

Knowledge of cervical cancer was assessed by asking the following questions.

• Are women with multiple partners at risk?

• Are women infected with HIV at risk?

• Are women who had sex before 18 years at risk?

• Are women with a family history of cervical cancer at risk? 

• Are women with husbands that have multiple partners at risk?

• What is the primary cause of cervical cancer?

• Are women with many children at risk?

The responses from the questions used to assess knowledge were scored, and the variable “knowledge of cervical cancer” was categorized based on these responses as “No correct response” if none of the responses were correctly answered and “at least one correct” if at least one response was correct.

Perceived susceptibility was determined by learning whether the patient thought she was at risk of contracting cervical cancer using the question, “Are you at risk of cervical cancer?” Participants who believed themselves to be at risk of cervical cancer were categorized as “high perceived susceptibility”, and those who believed otherwise were categorized as “low perceived susceptibility.”

*Perceived severity was assessed using the following questions*

• Is cervical cancer a threat to your emotional relationship?

• How long do you think you can live with HIV?

• How long do you think you can live with HIV and cervical cancer?

Participants who responded that they could live longer having only HIV infection rather than having both cervical cancer and HIV infection were categorized to have “high perceived severity” for cervical cancer. The severity level for subjects who thought otherwise or believed having cervical cancer does not reduce or increase their number of survival years were categorized as having “low perceived severity” for cervical cancer.

*Statistical analysis*

The data collected was transferred from the Kobo Toolbox-generated Excel spreadsheet into SAS 9.4 software for analysis. Age, number of live births, and income per month were collected as continuous variables, but they were included in the analysis as categorical variables. The summary statistics of socio-demographic variables, knowledge, perceptions, awareness, uptake and willingness of cervical cancer screening were displayed using frequencies and percentages. 

Awareness of cervical cancer, uptake of cervical cancer screening and willingness to be screened were included in three separate logistic regression models as outcome variables with the women’s sociodemographic variables as predictors. All sociodemographic variables obtained during the survey were included in the model during regression analysis. Variables with the highest p values were excluded from the model in a manual backward selection method. Variables with p values greater than 0.05 which did not affect other statistically significant variables where retained in the model. Model fit was examined using the Hosmer Lemeshow goodness of fit test. Interactions among the variables were checked using the Pearson chi-square test of a three-way contingency table. Confounders among the variables were checked by comparing the p-value of the likelihood ratio when testing the global null hypothesis and the p-value of the individual variables on the Type 3 table of the SAS output. This analysis did not demonstrate any evidence of collinearity. 

## Results

*Description of the study population*

A total of 421 women participated in the study, representing approximately 71% of the 593 female patients attending the HIV CTC at DRRH per month. This clinic is in the urban part of Dodoma thus most of the patients (n=331, 79%) were urban dwellers. The mean age of the population was 44.10 (standard deviation (SD) =10.65) years old. Average monthly income was $39.45 (US dollars, SD=8.9), and the majority (n=390, 93%) of the participants had no regular monthly income. About half of the participants had at least three live births (n=137, 35%), sexual intercourse at ages younger than 18 years (n=150, 36%), and reported that they were currently sexually active (50%, n=210) (see Figure 1A and 1B). The participant’s mean length of HIV infection since diagnosis was 5.4 (SD=4) years 

*Participant’s knowledge and perceptions of cervical cancer*

Seven questions were used to assess the patient’s knowledge of cervical cancer (Figure 2). Approximately 72% of the respondents did not know that having multiple sex partners increased cervical cancer risks. Only 11% of the participants knew that having HIV infection makes them at risk of developing cervical cancer. Almost all the participants did not know that having many children (97%) and having sex before 18 years old (94%) could increase the risk of cervical cancer. Almost all the participants (99%) did not know that the family history of cervical cancer can increase the risk of developing cervical cancer. Only 6% of the participants knew that women with a partner or husband who have multiple sex partners are at risk of cervical cancer. Only 7% of the participants were able to correctly identify the primary cause of cervical cancer.

The perceptions of the participants based on the belief of being at risk (perceived susceptibility) or the belief of being in danger (perceived severity) were also assessed. The majority (n=340, 81%) of the participants believed they were not at risk of cervical cancer. Most of the participants (n=265, 65%) did not believe having cervical cancer would threaten their emotional relationship (Table 1, Figure 2). 

*Factors associated with cervical cancer screening*

Educational level completed (p=0.01), income per month (p=0.02), age group (p<0.0001), and area of residence (p<0.0001) were all significantly associated to awareness of cervical cancer. Prior uptake of cervical cancer screening was significantly associated with the number of live births (p=0.001) and area of residence (p=0.04). Willingness to be screened was significantly associated with age groups (p=0.03) and the number of live births (p=0.03), Table 2.

*Awareness of cervical cancer screening*

To determine factors associated with awareness of cervical cancer among participants of this study, factors from table 2 were included in a logistic regression model. With the reduced model, it was found that only age groups (30-39 vs 18-29, OR=2.02 95% CI: 1.40-2.91, ≥40 vs. 18-29, OR=0.43, 95% CI: 0.31-0.61 and ≥40 Vs.30-39, OR=0.21 95% CI: 0.12-0.39) and area of residence (OR=3.68 95% CI: 2.12-6.38) were significantly associated with awareness of cervical cancer in this population. 

*Uptake of cervical cancer screening*

The odds of being screened at least once were 2% less for participants per 1-year increase in age (95% CI: 0.95-1.01). The odds of being screened for participants residing in the urban areas were 3.72 times higher than participants residing in rural areas (95% CI 1.05- 13.20). Participants with 1-2 live births had 2.52 times the odds of being screened compared to participants without any live births (95% CI: 1.14-5.56) but participants with at least 3 live births were 78% less likely to have been screened for cervical cancer when compared to participants with 1-2 live births (95% CI:0.09-0.57) – see Table 4. 

*Willingness to be screened*

Table 5 shows the likelihood of willingness to be screened among participants who had never been screened but were aware of cervical cancer. After adjusting for other variables, the odds of willingness to be screened were found to decrease for the participants who differ by one year (OR: 0.93 95% CI: 0.89-0.97).

**Figure 1A.**
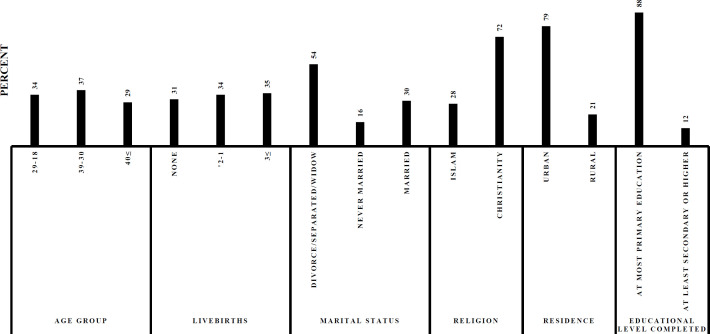
Descriptive Statistics of All the Participants in HIV CTC, DDRH, Dodoma, 2017 (N=421).

**Figure 1B:**
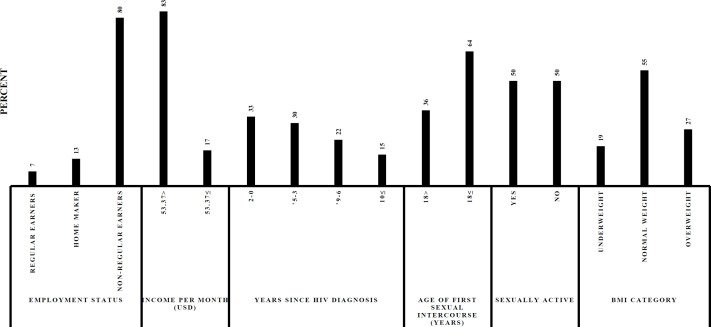
Descriptive Statistics of All the Participants in HIV CTC, DDRH, Dodoma, 2017 (N=421) Cont’d

**Table 1:** Participants’ Perceptions about Cervical Cancer (N=421)

Perceptions	Frequency	Percentage
Perceived susceptibility to cervical cancer		
Having risk of cervical cancer	78	19
Perceived severity of cervical cancer		
I can live longer with only HIV than if I have both HIV and cervical cancer	336	92
I can live longer with both HIV and cervical cancer than with only HIV	29	8
Missing	56	
Cervical cancer threatens my emotional relationship	142	35

**Figure 2:**
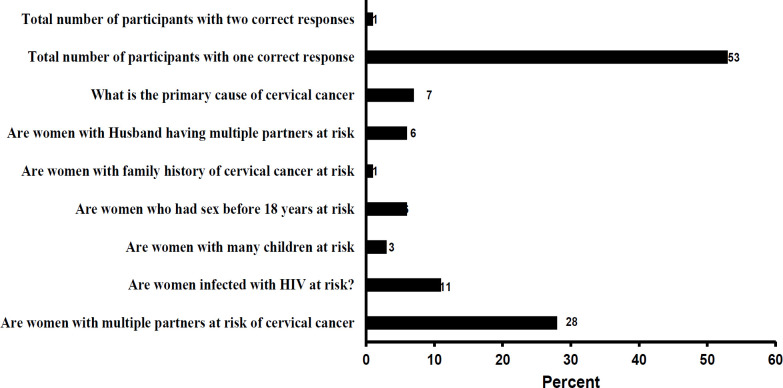
Knowledge Assessment for Cervical Cancer among Participants with Correct Response in HIV CTC (N=421)

**Figure 3:**
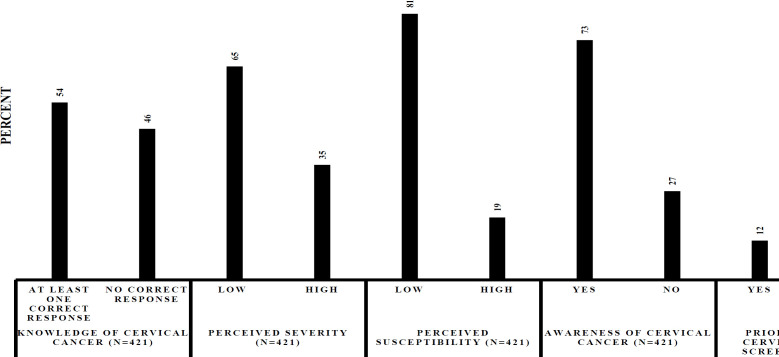
Summary of the Participants Attitude towards Cervical Cancer and Screening in HIV CTC

**Table 2:** Relationship between Participant Characteristics and Cervical Cancer Screening among Patients Receiving Antiretroviral Drugs in HIV CTC, DRRH 2017

Factors	Aware of cervical cancer N=306 (73%)	Not aware of cervical cancer N=113 (27%)	*P*-value	Screened at least once in their lifetime N=49 (16%)	Never screened N= 257 (84%)	*P*- value	Willing to screen N= 231 -91%	Not willing to screen N=24 (9%)	*P*- value
Age group									
18-29	110 (36)	32 (28)		18 (37)	92 (36)		89 (39)	3 (13)	
30-39	127 (42)	29 (26)	<0.0001	24 (49)	103 (40)	0.29	90 (39)	12 (50)	0.03
≥40	68 (22)	52 (46)		7 (14)	61 (24)		51 (22)	9 (37)	
Missing	1	-		-	1		1	-	
Livebirths									
None	90 (31)	32 (31)		12 (26)	78 (32)		67(31)	10(48)	
1-2	105 (36)	28 (27)	0.1	27 (59)	78 (32)	0.001	69 (31)	2 (10)	0.03
≥3	94 (33)	43 (42)		7 (15)	87 (36)		84 (38)	9 (42)	
Missing	17	10		3	14		11	3	
Marital status									
Once married	160 (52)	67 (59)		27 (55)	133 (52)		118 (51)	14 (58)	
Never married	52(17)	16 (14)	0.4	9 (18)	43 (16)	0.78	40 (17)	3 (13)	0.8
Married	94(31)	30 (27)		13 (27)	81 (32)		73 (32)	7 (29)	
Missing	-	-		-	-		-	-	
Religion									
Islam	87 (28)	27 (24)		18 (37)	69 (27)		61 (26)	8 (33)	
Christianity	219 (72)	86 (76)	0.4	31 (63)	188 (73)	0.16	170 (74)	16 (67)	0.5
Missing	-	-		-	-		-	-	
Residence									
Urban	256 (84)	74 (66)	<0.0001	46 (94)	210 (82)	0.04	191 (83)	18 (75)	0.4
Rural	50 (16)	39 (34)		3 (6)	47 (18)		40 (17)	6 (25)	
Missing	-	-		-	-		-	-	
Educational level completed									
At most primary education	263 (86)	107 (95)	0.01	40 (82)	223 (87)	0.34	201 (32)	20 (83)	0.6
At least secondary or higher	43(14)	6(5)		9 (18)	34 (13)		30 (68)	4 (17)	
Missing	-	-		-	-		-	-	
Employment Status									
Regular earners	25 (8)	6 (5)	0.5	6 (12)	19 (7)	0.28	16 (7)	3 (13)	0.3
Home maker	37 (12)	17 (15)		8 (16)	29 (11)		28 (12)	20 (83)	
Non-regular earners	244 (80)	90 (80)		35 (72)	209(81)		187(81)	1(4)	
Missing	-	-		-	-		-	-	
Income per month (USD)									
<53.37	242 (80)	100 (90)	0.02	36 (75)	206 (81)	0.33	185 (81)	19 (79)	0.8
≥53.37	60 (20)	11 (10)		12 (25)	48 (19)		43 (19)	5(21)	
Missing	4	2		1	3		3	-	
Years since HIV diagnosis									
0-2	95 (31)	43 (38)		17 (35)	78 (31)		71 (31)	6 (25)	
3-5	86 (28)	39 (35)	0.08	12 (25)	74 (29)	0.85	68 (30)	5 (21)	0.5
6-9	74 (24)	17 (15)		11 (22)	63 (25)		56 (25)	7 (29)	
≥10	49 (16)	13 (12)		9 (18)	40 (16)		34 (14)	6 (25)	
Missing	2	1		-	2		2	-	

**Table 3:** Likelihood of Women Receiving ARVs in DRRH to be aware of Cervical Cancer (N=421)

Characteristics	Crude odds ratio	95% Confidence Interval		Adjusted odds ratio	95% Confidence Interval		Reduced Model		
							Odds ratio	95% Confidence Interval	
		Lower	Upper		Lower	Upper		Lower	Upper
Age group									
30-39 Vs. 18-29	1.78	1.26	2.52	2.02	1.38	2.96	2.02	1.4	2.91
≥ 40 Vs. 18-29	0.46	0.33	0.63	0.43	0.29	0.62	0.43	0.31	0.61
≥ 40 Vs.30-39	0.26	0.14	0.46	0.21	0.11	0.41	0.21	0.12	0.39
Years since HIV diagnosis									
3-5 Vs. 0-2	0.78	0.54	1.15	0.77	0.51	1.18			
6-9 Vs. 0-2	1.39	0.89	2.17	1.39	0.86	2.26			
≥ 10 Vs 0-2	1.26	0.75	2.1	1.42	0.79	2.54			
6-9 Vs. 3-5	1.77	0.91	3.44	1.79	0.87	3.73			
≥ 10 Vs 3-5	1.6	0.75	3.4	1.84	0.78	4.33			
≥ 10 Vs 6-9	0.91	0.4	2.06	1.02	0.41	2.54			
Area of Residence									
Urban Vs. Rural	3.12	1.87	5.2	4.02	2.26	7.14	3.68	2.12	6.38
Livebirths									
1-2 Vs. None	1.32	0.94	1.84	1.38	0.96	2.95			
≥ 3Vs. None	0.75	0.55	1.03	0.9	0.63	1.28			
≥ 3 Vs. 1-2	0.57	0.33	0.99	0.65	0.35	1.21			
Educational level completed									
at least secondary or higher Vs. at most primary education	2.22	0.91	5.46	1.31	0.48	3.6			
Monthly Income (USD)									
≥ 53.37 Vs **<** 53.37	2.14	1.04	4.38	1.72	0.79	3.77			

**Table 4:** Factors Associated with Prior Uptake of Cervical Cancer Screening at Least Once in Their Lifetime among Participants who are Aware of Cervical Cancer (N=306)

Factors	Crude OR	95% Confidence Interval		Adjusted OR	95% Confidence Interval		Reduced Model		
							Odds ratio	95% Confidence Interval	
		Lower	Upper		Lower	Upper		Lower	Upper
Age (Per 1 year increase)	0.98	0.95	1.01	0.98	0.94	1.02			
Years since HIV diagnosis (per 1-year increase)	0.99	0.91	1.07	1.02	0.93	1.11			
Religion									
Islam vs. Christian	1.58	0.83	3	1.4	0.69	2.85			
Area of Residence									
Urban vs. Rural	3.43	1.02	11.51	3.72	1.05	12.5	3.59	1.04	12.4
Livebirths									
1-2 vs. None	2.25	1.06	4.76	2.52	1.16	5.47	2.46	1.15	5.25
≥ 3 vs. None	0.52	0.2	1.4	0.55	0.17	1.39	0.47	0.17	1.33
≥3 vs. 1-2	0.23	0.1	0.56	0.22	0.07	0.5	0.19	0.08	0.5

**Table 5:** Likelihood of Women Receiving ARVs in DRRH to be Willing to Screen for Cervical Cancer among Those who have Never been Screened (N=257)

Factors	Crude OR	95% Confidence Interval		Adjusted OR	95% Confidence Interval		Reduced model		
							Odds ratio	95% CI	
		Lower	Upper		Lower	Upper		Lower	Upper
Age (Per 1-year increase)	0.93	0.89	0.97	0.93	0.88	0.97	0.93	0.89	0.97
Years since HIV diagnosis (Per 1-year increase)	0.96	0.87	1.06	1.01	0.9	1.14			
Area of Residence									
Urban vs. Rural	1.09	0.35	3.41	1.08	0.32	3.65			

## Discussion

Cervical cancer is one of the main causes of morbidity and mortality among women (Sopian et al. 2019) and WLWHIV have increased risk of cervical cancer (Kelly et al., 2018). That is why it is paramount to explore the level of awareness, knowledge, uptake, and willingness to screen for cervical cancer in this population. 

Participation in this study was relatively high, with a response rate of 71% at HIV CTC in DRRH Dodoma. Similar studies in HIV clinics across Africa have received high response rates. The response rate of the studies in Nigeria was 91% (Ezechi, et al., 2013); in Ethiopia, 97% (Belete et al., 2015); and 100% in Durban, South Africa (Ports et al., 2015). This indicates that the WLWHIV in sub-Saharan Africa are willing to participate in studies related to the improvement of their health. This participatory attitude observed among WLWHIV may suggest high receptivity to health education and research involving public health issues. HIV clinic administrators could take this opportunity to introduce additional programs to positively impact the health outcomes of WLWHIV.

The level of awareness of cervical cancer was high (73%) in this study, with the media representing the most frequently cited (76%) source of information. The media was also found to be the prevailing source of information for cervical cancer in a similar study carried out in Dar es Salaam (Koneru et al., 2017). Given the uptake of cervical cancer screening is low (16%) despite higher proportion of awareness, it may be safe to conclude that the lack of screening may be due to poor knowledge of cervical cancer among women at HIV CTC. Information publicized by the media about cervical cancer may be inadequate or misleading thereby resulting in poor attitude towards uptake of cervical cancer screening. Most of the women (89%) in this study did not believe that WLWHIV have higher risk of developing cervical cancer, and many (93%) did not know the primary cause of cervical cancer. Neither did most of the women (72%) believe that having multiple partners increases the risk of cervical cancer nor did they know that women who have a family history of cervical cancer are at risk (99%). Based on these results, we support the suggestion that knowledge of cervical cancer may be different from awareness (Shiferaw et al., 2016). 

Findings from other studies reported that most WLWHIV in both developed and underdeveloped countries do not perceive themselves to be at risk of cervical cancer (Kenya et al., 2015; Lambert et al., 2015; Williams et al., 2015; Koneru et al., 2017). Similarly, in this current study, we showed that 81% do not perceive to be at risk of cervical cancer. This points to the lack of proper education on the risk of cervical cancer among HIV-infected women. 

In this study, we explored the different socio-demographic factors that may be associated with awareness and uptake of cervical cancer. We found a significant association between age and awareness of cervical cancer. Women aged 30-39 years were found to be two times more likely to have heard of cervical cancer when compared to women aged 18-29 years. Uptake of cervical cancer screening was found to decrease with advancing age. In contrast, findings reported in the studies conducted in Kenya (Rosser et al., 2015), Ethiopia (Belete et al., 2015) and Nigeria (Ezechi et al., 2013) revealed that uptake of screening increased with age. This maybe due to differences in settings. As the 30-39 age range is representative of reproductive age for most women in a resource-limited environment, the high awareness of cervical cancer screening might be due to more frequent exposures to reproductive care and services than women in other age groups. 

Findings from this study suggest that women residing in urban areas were more likely to be aware and uptake cervical cancer screening as compared to rural dwellers. The disparity in terms of awareness of cervical cancer between rural and urban women at the HIV CTC validates the media as the primary source of information on cervical cancer instead of the HIV CTC. The study also shows that if the HIV CTC, which is the meeting point for care delivery for both rural and urban WLWHIV was the primary source of information on cervical cancer, the level of awareness for both rural and urban WLWHIV was the same. This is not surprising as it addresses the issue of access to information on cervical cancer which might have placed the urban women at an advantage. The gap in access to information can be bridged by providing necessary and adequate information to women at the HIV CTC. Beyond access and availability of screening, the potential gap - in the attitude to screen and knowledge by urban versus rural dwellers is another issue that requires additional attention (Nwankwo et al., 2011). 

In this study, the uptake of cervical cancer screening by HIV positive women was 16% between 2014 and August 2017. This result is similar to the screening rates reported by studies conducted in Dar es Salaam at 9%, (Koneru et al., 2017), Tanzania’s Kilimanjaro region at 6% (Cunningham et al., 2015), Nigeria at 9% (Ezechi et al.,2013), Addis Ababa at 12% (Belete et al., 2015), 4.8% in Uganda (Ndejjo et al., 2016) and in northwest Ethiopia at 10% (Nega et al., 2018). These figures emphasize the need for cervical cancer screening among HIV women in many HIV clinics in Sub Sahara Africa. South Africa (Maree and Moitse, 2014) and western Kenya (Rosser et al., 2015) have reported a relatively elevated screening rate at 32% and 84%, respectively, which may be due to consistent education and access and availability of free screening services in these areas. Such programs and services are missing at HIV CTC in Dodoma. 

The willingness to screen for cervical cancer among women who reported to have never screened for cervical cancer was 90% in this study. Lower prevalence of women indicating willingness to screen were reported in studies conducted in Nigeria at 80% (Ezechi et al., 2013) Ethiopia at 62.7% (Belette et al.,2015), Ghana at 82% (Ebu and Ogah, 2018) and Kenya at 44% (Njuguna et al., 2017). Despite the low uptake of cervical cancer screening observed in our findings, the high prevalence of willingness to screen among women at HIV CTC speaks volume about the success that would be achieved if cervical cancer screening is integrated to HIV care delivery services. Previous studies reported that older age was associated with willingness to screen (Ndejjo et al., 2017; Ezechi et al., 2013; Belete et al., 2015; Njuguna et al., 2017). In contrast to these findings, we found that younger age at HIV CTC is significantly associated with willingness to screening. Although the risk of cervical cancer is high among HIV women, it is even higher among older women as compared to younger women (Diver et al., 2018). It is therefore important to promote screening awareness and opportunity to older women who may be unwilling to screen for cervical cancer at HIV CTC given that this is a resource-limited setting. Unlike other similar studies, we included geographic characteristics in our model to determine the impact of residential location on the willingness to screen. We found that participants residing in urban areas were more willing to be screened when compared to their counterparts in rural areas. However, this study did not investigate the reasons why urban dwellers would be more willing to screen compared to rural dwellers hence the need for additional study. 

This study was strengthened by the presence of limited missing observations. Furthermore, the use of female interviewers who were staffs of the hospital allowed the patient’s full disclosure of all required information. This study identified gaps with respect to education and awareness about cervical cancer which was possible because the research team constitutes members of the care team in the clinic. Although the media in Dodoma have proven to reliably reach out on cervical cancer, better education can only be accomplished by the health system. It is understandable that the high patient-to-staff ratio at the HIV CTC might not allow adequate time for educating patients on cervical cancer, but posters on the walls of the HIV clinic and other educational materials given to the patients at visits could be a good strategy to improve in this area (Khozaim et al., 2014)

A limitation of this study was interviewer bias. It is possible that some of the participant’s responses might have been influenced by the body language or tone of the interviewers despite interviewer training. Also, some of the participants who have adequate knowledge of mobile phones answered the survey themselves while others were helped by the research assistants. This could impact our results because we did not track if there was a difference between the method of survey administration. 

It is therefore recommended that future studies allow a more comprehensive approach to understanding cervical cancer screening at HIV CTC, DRRH Dodoma, by including the care team in the survey to determine potential barriers to uptake of cervical cancer screening from providers’ perspectives.

In conclusion, our findings showed that despite demonstrated awareness of cervical cancer, knowledge about risk of cervical cancer and uptake of cervical cancer screening was low, although willingness to screen was high among the study population. We also showed factors associated with these outcomes, including age and geography of residence. Younger age was found to be associated with the willingness to screen. Although the willingness to screen was higher among urban women, it was not statistically significant. Our findings do not provide a definite conclusion in regard to the factors associated with awareness, uptake, and willingness to screen for cervical cancer in HIV CTC. If larger samples were used for this study, other factors which were not associated might become significantly associated with willingness or uptake of cervical cancer screening. Most of the participants in this study were found to lack the perceived susceptibility or severity of cervical cancer. Education is therefore needed for women to understand their risks of developing cervical cancer and how severe it is especially among WLWHIV. Meanwhile, older participants who identified as unwilling to screen for cervical cancer should be considered for specific education and other health promotion activities. 

## Ethical issues

Approval for this study was obtained from the University of Nebraska Medical Center and University of Dodoma Review Board. Informed consent was written and signed by all participants before data was collected, and all data were stored and analysed on an encrypted electronic device.

## References

[B1] Afri-Dev.Info, Information &amp; Analysis on Health, Population, Human &amp; Social Development (2014). Africa Cervical Cancer Multi-Indicator Incidence &amp; Mortality Scorecard.

[B2] Bansil P, Lim J, Byamugisha J (2015). Performance of cervical cancer screening techniques in HIV-infected women in Uganda. J Low Genit Tract Dis.

[B3] Belete N, Tsige Y, Mellie H (2015). Willingness and acceptability of cervical cancer screening among women living with HIV/AIDS in Addis Ababa, Ethiopia: a cross-sectional study. Gynecol Oncolo Res Pract.

[B5] Biesma RG, Brugha R, Harmer A (2009). The effects of global health initiatives on country health systems: a review of the evidence from HIV/AIDS control. Health Policy Plann.

[B6] Champman CL, Harris AL, Enah C (2016). Confusion about HPV and cervical cancer among Black/African-American women living with HIV. J Natl Black Nurses Assoc.

[B7] Cunningham MS, Skrastins E, Fitzpatrick R (2015). Cervical cancer screening and HPV vaccine acceptability among rural and urban women in Kilimanjaro Region, Tanzania. BMJ Open.

[B8] Del Carmen MG, Rice LW, Schmeler KM (2015). Global health perspective on gynecologic oncology. Gynecol Oncol.

[B9] Diver EJ, Hinchcliff EM, Gockley AA (2018). Assessment of treatment factors and clinical outcomes in cervical cancer in older women compared to women under 65 years old. J Geriatr Oncol.

[B10] Ebu NI, Ogah JK (2018). Predictors of cervical cancer screening intention of HIV-positive women in the central region of Ghana. BMC Womens Health.

[B11] Ezechi OC, Gab-Okafor CV, Ostergren PO, Pettersson KO (2013). Willingness and acceptability of cervical cancer screening among HIV positive Nigerian women. BMC Public Health.

[B12] Ezechi OC, Pettersson KO, Okolo CA, Ujah IA O, Ostergren PO (2014). The association between HIV infection, antiretroviral therapy and cervical squamous intraepithelial lesions in South Western Nigerian women. PLoS One.

[B13] Gaffing S, Gupta N (2016). Cervical cancer screening in HIV positive women. Eur J Surg Oncol.

[B14] Getahun F, Mazengia F, Abuhay M, Birhanu Z (2013). Comprehensive knowledge about cervical cancer is low among women in Northwest Ethiopia. BMC Cancer.

[B16] Grellier N, Quéro L (2014). Cervical cancer: particularities in HIV patients. Bull Du Cancer.

[B18] Jedy-Agba E, Joko WY, Liu B (2020). Trends in cervical cancer incidence in sub-Saharan Africa. Br J Cancer.

[B19] Kafuruki L, Rambau PF, Massinde A, Masalu N (2013). Prevalence and predictors of cervical intraepithelial neoplasia among HIV infected women at Bugando Medical Centre, Mwanza-Tanzania. Infect Agent Cancer.

[B20] Kelly H, Weiss HA, Benavente Y (2018). Association of antiretroviral therapy with high-risk human papillomavirus, cervical intraepithelial neoplasia, and invasive cervical cancer in women living with HIV: a systematic review and meta-analysis. Lancet HIV.

[B21] Kenya S, Carrasquillo O, Fatil M (2015). Human papilloma virus and cervical cancer education needs among HIV-positive Haitian women in Miami. Womens Health Issues.

[B22] Khozaim K, Orang’o E, Christoffersen-Deb A (2014). Successes and challenges of establishing a cervical cancer screening and treatment program in western Kenya. Int J Gynecol Obstet.

[B23] Koneru A, Jolly PE, Blakemore S (2017). Acceptance of peer navigators to reduce barriers to cervical cancer screening and treatment among women with hiv infection in Tanzania. Int J Gynecol Obstet.

[B24] Kumakech E, Andersson S, Wabinga H, Berggren V (2015). Integration of HIV and cervical cancer screening perceptions and preferences of communities in Uganda. BMC Womens Health.

[B25] Lambert CC, Chandler R, McMillan S (2015). Pap test adherence, cervical cancer perceptions, and HPV knowledge among HIV-infected women in a community health setting. J Assoc Nurses AIDS Care.

[B26] Mukama T, Ndejjo R, Musabyimana A, Halage AA, Musoke D (2017). Women’s knowledge and attitudes towards cervical cancer prevention: a cross sectional study in Eastern Uganda. BMC Womens Health.

[B27] Maree JE, Moitse KA (2014). Exploration of knowledge of cervical cancer and cervical cancer screening amongst HIV-positive women. Curationis.

[B28] Mboumba Bouassa RS, Prazuck T (2017). Cervical cancer in sub-Saharan Africa: a preventable noncommunicable disease. Expert Rev Anti Infect Ther.

[B30] Ndejjo R, Mukama T, Musinguzi G (2017). Women’s intention to screen and willingness to vaccinate their daughters against cervical cancer–a cross sectional study in eastern Uganda. BMC Public Health.

[B31] Ndejjo R, Mukama T, Musabyimana A, Musoke D (2016). Uptake of cervical cancer screening and associated factors among women in rural Uganda: a cross sectional study. PLoS One.

[B32] Nega AD, Woldetsadik MA, Gelagay AA (2018). Low uptake of cervical cancer screening among HIV positive women in Gondar University referral hospital, Northwest Ethiopia: cross-sectional study design. BMC Womens Health.

[B33] Njuguna B, Vorkoper S, Patel P (2018). Models of integration of HIV and noncommunicable disease care in sub-Saharan Africa: lessons learned and evidence gaps. AIDS.

[B34] Nwankwo KC, Aniebue UU, Aguwa EN, Anarado AN, Agunwah E (2011). Knowledge attitudes and practices of cervical cancer screening among urban and rural Nigerian women: a call for education and mass screening. Eur J Cancer Care.

[B35] Ports KA, Haffejee F, Mosavel M, Rameshbabu A (2015). Integrating cervical cancer prevention initiatives with HIV care in resource-constrained settings: a formative study in Durban, South Africa. Global Public Health.

[B36] Redfield RR, Modi S, Moore CA (2019). Health care autonomy of women living with HIV. N Engl J Med.

[B37] Rohner E, Bütikofer L, Schmidlin K (2020). Cervical cancer risk in women living with HIV across four continents: a multicohort study. Int J Cancer.

[B38] Rosser JI, Njoroge B, Huchko MJ (2015). Cervical cancer screening knowledge and behavior among women attending an urban hiv clinic in western Kenya. J Cancer Edu.

[B39] Shiferaw N, Brooks MI, Salvador-Davila G (2016). Knowledge and awareness of cervical cancer among HIV-infected women in Ethiopia. Obstet Gynecol Int.

[B40] Sichanh C, Fabrice QUET, Chanthavilay P (2014). Knowledge, awareness, and attitudes about cervical cancer among women attending or not an HIV treatment center in Lao PDR. BMC Cancer.

[B41] Sopian MM, Din SA T, Hussin H (2019). Obstacles to implementing the HPV vaccine: Is it worth pursuing or not?. Asian Pac J Cancer Care.

[B42] Somi G, Matee M, Makene CI (2009). Three years of HIV/AIDS care and treatment services in Tanzania: achievements and challenges. Tanzania J Health Res.

[B44] Vaccarella S, Lortet-Tieulent J, Plummer M, Franceschi S, Bray F (2013). Worldwide trends in cervical cancer incidence: impact of screening against changes in disease risk factors. Eur J Cancer.

[B45] Williams M, Moneyham L, Kempf MC, Chamot E, Scarinci I (2015). Structural and sociocultural factors associated with cervical cancer screening among HIV-infected African American women in Alabama. AIDS Patient Care STDS.

[B46] World Health Organization (WHO) (2000). Comprehensive cervical cancer control; A guide to essential practice.

